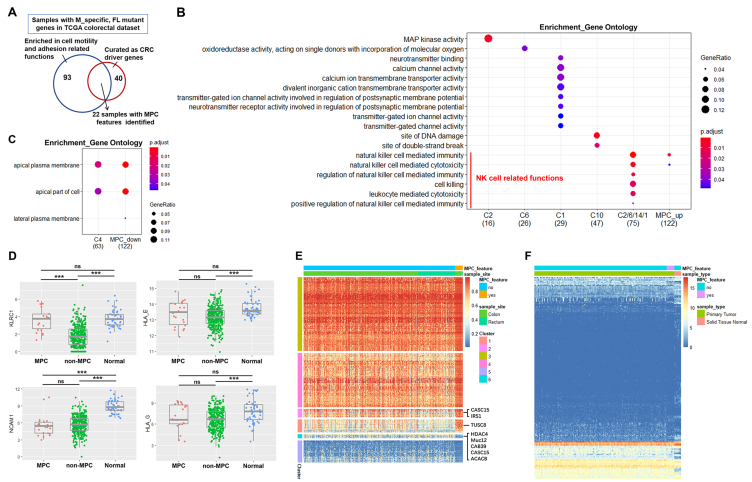# Investigation of the genome-wide signatures underlying the micropapillary carcinoma components in colorectal cancers

**DOI:** 10.1016/j.gendis.2024.101331

**Published:** 2024-05-19

**Authors:** Yingying Meng, Zhao Zhang, Tao Tang, Yanhong Yu, Dan Wang, Wei Sun

**Affiliations:** a School of Medicine, Nankai University, Tianjin 300071, China; b Department of Anorectal, The Nankai University Affiliated Union Medical Center, Tianjin 300131, China; c Department of Pathology, The Nankai University Affiliated Union Medical Center, Tianjin 300131, China; d Department of Pathology, Tianjin Medical University General Hospital, Tianjin 300052, China

The invasive micropapillary carcinoma (MPC) is a rare and specialized pathologic subtype of cancer. MPCs are highly aggressive, highly metastatic, and commonly associated with poor prognosis. MPC components have been found at least in breast, bladder, lung, ovary, parotid gland, stomach, and colorectum tumors. Morphologically, MPCs typically consist of small clusters of neoplastic cells with distinct polarity reversal and tight adhesion with each other, usually situating in the tumor invasion front.^1^ However, pure MPC tumors are extremely rare, MPCs commonly co-exist with other pathological fractions, such as adenocarcinoma, in tumor entity, and their proportion generally ranges from 5% to 95%.^1^ Although highly malignant, knowledge concerning the mechanisms underlying MPC behavior and critical events driving tumor progression toward MPC remains rather limited. Recently, two reports indicate that the copy-number loss of IGSF9 (immunoglobulin superfamily 9) and PRDM16 (PR domain containing 16) and copy-number gain of ALDH2 (aldehyde dedydrogenase-2) in breast MPCs are associated with high metastatic potential^2^ and that the aberrant activity of RhoA plays a critical role in polarity-switching in colorectal MPCs,^3^ respectively.

Aiming to further investigate the additionally genome-wide characteristics of colorectal MPCs, we firstly incorporated a panel of 10 colorectal cancer patients carrying typical MPC fractions, isolated the paired MPC, adenocarcinoma, and paracancerous components from each patient's formalin-fixed paraffin-embedded tumor tissue by micro-perforating and subjected all these samples to whole genome sequencing (Fig. 1A and Table S1). In general, the distribution of somatic single nucleotide variations (SNVs) and small insertion-deletions (Indels) on different annotated genomic regions (Fig. S1A, B), the mutational signatures (Fig. S1C, D), and the copy number variations (Fig. S2A–C) showed considerable similarities, particularly between paired MPC (M) and adenocarcinoma (A) samples, suggesting the probably common initiation and shared early tumor-driving events of the commensal MPCs and adenocarcinomas.

**Figure 1:**
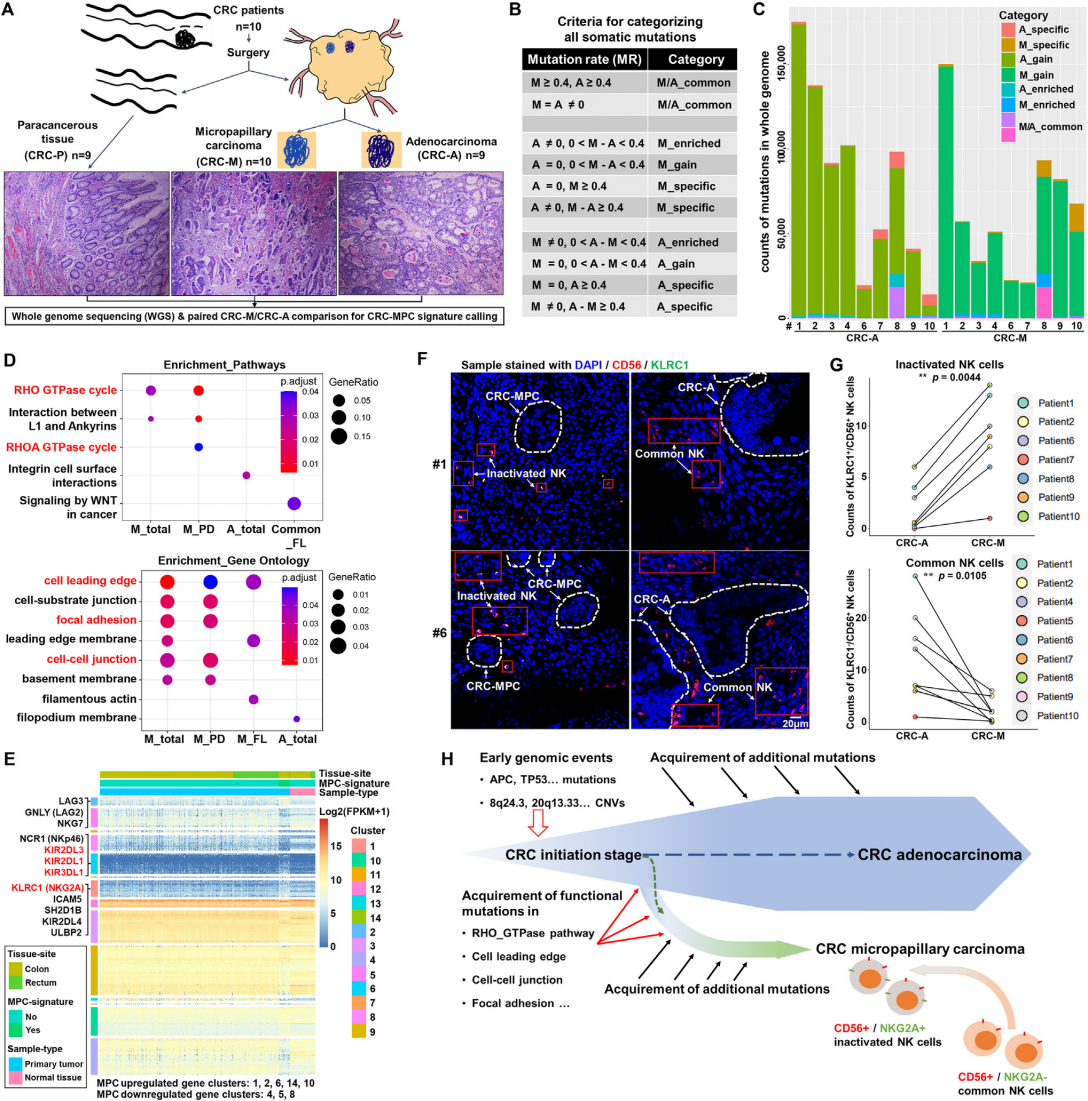
The investigation of the characteristics of micropapillary carcinoma (MPC) components in colorectal cancers. **(A)** A sketch map describing the overall design of this study. Components of colorectal micropapillary carcinoma (CRC-M), adenocarcinoma (CRC-A), and paracancerous tissue (CRC-P) were subjected to whole genome sequencing and for each patient, CRC-P was used as the matched normal sample for somatic mutation calling of paired CRC-M and CRC-A samples. Representative hematoxylin-eosin staining images of paired CRC-P, CRC-M, and CRC-A from the sample cohort in this study were also shown. **(B)** The strategy of distinguishing the divergencies of somatic mutations between MPC and adenocarcinoma components. The mutational rate (MR) = 0.4 was set as a threshold and the criteria for defining the mutation categories of M_specific, A_specific, M_gain, A_gain, M_enrich, A_enrich, and M/A_common were described in table. **(C)** The distribution of mutations in the whole-genome of each sample in M_specific, A_specific, M_gain, A_gain, M_enrich, A_enrich, and M/A_common categories. Data were given in a stacked bar plot. M, micropapillary carcinoma; A, adenocarcinoma. **(D)** The results of pathway (top) and gene ontology (bottom) enrichment assays performed with mutant genes in each category as marked on the plots. Data were given in dot plots and relative information was given by the legends. M_total, total MPC-specific coding mutations; M_PD, MPC-specific possibly-damaged mutations; M_FL, MPC-specific function-loss mutations; A_total, total adenocarcinoma-specific coding mutations; common_FL, function-loss mutations in M/A_common group. **(E)** Clustering of the top 500 differentially expressed genes (DEGs) between the groups of samples with or without MPC signature (MPC-signature_yes and _no groups) in TCGA colorectal cancer dataset. Genes were clustered into 14 groups as indicated on the plot, and typical DEGs (inactive natural killer/NK cell marker genes in red) were marked on the plot. Other information was given by the legends. **(F)** Representative images of immunofluorescent (IF) staining (from patient #1 and #6) showing the status of NK cells in paired MPC and adenocarcinoma samples. KLRC1 and CD56 antigen were labeled with fluor-488 and fluor-546 fluorescence, respectively. The nuclei were stained with DAPI. Relative information was marked on the plots. **(G)** Quantification of the immunofluorescent staining results in the incorporated colorectal cancer samples in this study. The amount of KLRC1^+^/CD56^+^ NK cells (inactivated NK cells) and KLRC1^−^/CD56^+^ NK cells (common NK cells) were counted from the images of each paired MPC and adenocarcinoma samples and paired *t*-test was adopted for statistical analysis. ∗*P* < 0.05, ∗∗*P* < 0.01. **(H)** A sketch map summarizing the tumor progression and the acquisition of the micropapillary carcinoma characteristics in colorectal cancers. Critical events during colorectal MPC progression and characteristics concerning to MPCs in the current study were shown in the plot.

Somatic mutation rate (MR) can reflect the timing of mutation events. Based on a MR threshold of 0.4, we categorized all the detected mutations into several different groups (Fig. 1B). Detailly, the M/A_specific groups, which represented the mutations acquired as early as at time of M/A division, were defined by the mutations specifically detected in MPC (M) or adenocarcinoma (A) components with MR ≥ 0.4, or identical mutations in paired components with the difference of MR ≥ 0.4; the M/A_gain groups were defined by the mutations specifically detected in M/A components with MR < 0.4, indicating that only partial M/A cells developed such mutations at later stages; in paired M/A components, identical mutations with the difference of MR < 0.4 were defined as M/A_enriched while those with equal MR or MR ≥ 0.4 in both were defined as M/A_common. It could be observed that the M/A_gain mutations commonly accounted for the majority in genome-wide among the samples (Fig. 1C). Further, coding region mutations were categorized into function-loss (FL) group, including stop-gain SNVs and frameshift Indels, and possibly-damaged (PD) group, including non-frameshift Indels and stop-loss and nonsynonymous SNVs. Notably, total MPC-specific coding mutations (M_total, including both FL and PD mutations in M_specific and M_gain groups) and MPC-specific_possibly-damaged mutations (M_PD) were specifically enriched on RHO-GTPase cycle (Fig. 1D; Fig. S3A), the pathway closely associated with colorectal MPC.^3^ Meanwhile, the function-loss mutations in M/A_common group (common_FL) were specifically enriched on WNT signaling pathway (Fig. 1D; Fig. S3B), a widely-accepted tumor-initiation pathway in colorectal cancers.^4^ In contrast, mutations in adenocarcinoma-specific (A_specific) and M/A_enriched groups lacked much specific enrichment (Fig. 1D), suggesting a probably more divergent mutational spectrum during the progression of common colorectal adenocarcinomas. Moreover, the MPC-specific_total, _possibly-damaged, and _function-loss mutations (M_total, M_PD, and M_FL) were additionally enriched on a series of cell adhesion and cell motility related functions annotated in Gene Ontology, including cell leading edge/leading edge membrane, focal adhesion, and cell–cell junction (Fig. 1D; Fig. S3C–E), all of which potentially contributed to the highly invasive performance of MPC.

Since the MPC signature could be defined by the gain of function-loss mutations of genes enriched on the above-mentioned functions, we next investigated the TCGA colorectal carcinoma cohort following this cue. 22 samples carrying both function-loss mutations in the above gene list (Table S2) and function-loss mutations in MPC_specific-FL gene list that were also curated as driver genes in colorectal cancers^5^ (Table S3) were categorized as the MPC-signature group (Fig. S4A). Top 500 differentially expressed genes between MPC-signature and non-MPC groups were subjected to clustering, and several clusters contained typical MPC-up-regulated or MPC-down-regulated genes were identified (Fig. 1E). Notably, the MPC-up-regulated genes could be specifically enriched on natural killer cell (NK cell) related functions (Fig. S4B), and the enriched genes in that group were primarily inactive NK cell markers, such as KIR2DL3, KIR2DL1, KIR3DL1, and particularly, KLRC1 (NKG2A) (Fig. 1E), strongly suggesting an increase of inactivated NK cells in these tumors. Meanwhile, the MPC-down-regulated genes were enriched on the terms of apical plasma membrane and apical part of cell (Fig. S4C), which could be reasonable for the polarity reversal of MPCs. Although KLRC1 level was prominently higher in MPC group than in non-MPC group, the level of its ligand genes, HLA-E and HLA-G, and the common NK cell marker gene NCAM1 (CD56) showed no prominent difference between the two groups (Fig. S4D), implying a switching from active to inactive status of NK cells in the MPC microenvironment. Accordingly, immunofluorescent staining on the same samples subjected to whole genome sequencing revealed that statistically, there were indeed more KLRC1^+^/CD56^+^ NK cells (inactivated NK cells) in the stroma around the MPC components, whereas the KLRC1^−^/CD56^+^ NK cells (common/active NK cells) were more enriched in the concomitant adenocarcinoma regions (Fig. 1F, G). Meanwhile, the methylation and miRNA expression profiles were analyzed as well, but no prominent features in these two levels were found in the MPC-signature group (Fig. S4E, F).

Although highly malignant, studies on MPC is a relatively less, particularly about the understandings of its initiation, critical driven events, and mechanisms underlying its biological behavior.^1^, ^2^, ^3^ Herein our investigation in colorectal MPCs addressed several key points concerning to these issues (Fig. 1H). Firstly, commensal MPC and adenocarcinoma components commonly shared largely identical genome-wide mutational signatures (*e.g.*, DNA repair abnormality), colorectum-preferred tumor driven mutations (*e.g.*, mutations in WNT and p53 signaling pathways), and copy number variations, suggesting the co-origination and co-development of MPC with other pathologic components at the initiation/early stage of tumorigenesis. Secondly, the diverging of MPC and other components such as adenocarcinoma from initial tumor seems to occur at a not-too-late stage during tumor progression, for that most somatic SNVs and Indels accumulated in MPC or adenocarcinoma components were specific (belonging to M/A_specific or M/A_gain groups), despite of their highly similar mutation signature profiles. Thirdly, the acquisition of extra gene mutations involved in the regulation of cell junction and cell motility, such as RHO-GTPase cycle, cell leading edge, and focal adhesion, seems to be essential to push forward the tumor progression towards MPC direction. The extensive accumulation of RHO-GTPase related mutations in MPC components is pivotal, if not prerequisite, for the dysfunction of RHO signaling pathway and therefore polarity reversal in colorectal MPCs.^3^ Finally, infiltration of inactivated NK cells was significantly increased in MPC fractions, seemingly due to the status switching of NK cells in MPC microenvironment. This may impair the tumor surveillance of NK cells and further accelerate the invasion and metastasis of MPC cells. Altogether, these findings shed new lights on the recognition of colorectal MPCs, and may bring about novel insights concerning to MPC diagnosis and treatment.

## Ethics declaration

The study was approved by the medical ethics committee of Nankai University affiliated Union Medical Center (approval number: 2014KZ058). Informed consent from the patients was obtained. The procedures involving human subjects were in accordance with the Helsinki Declaration of 1975.

## Conflict of interests

There is no conflict of interests concerning this work.

## Funding

This work was funded by the National Natural Science Foundation of China (No. 32370820, 81772976), Tianjin Science and Technology Funding (China) (No. 21JCYBJC00300), and the Key Project of Traditional Chinese Medicine of Tianjin Health Commission (No. 2022006).

## Data availability

All sequencing data in this study in fastq format were available at the China National Center for Bioinformation (CNCB, https://ngdc.cncb.ac.cn/hgrip/, accession number: HRA003804).

## Figures and Tables

**figs1:**
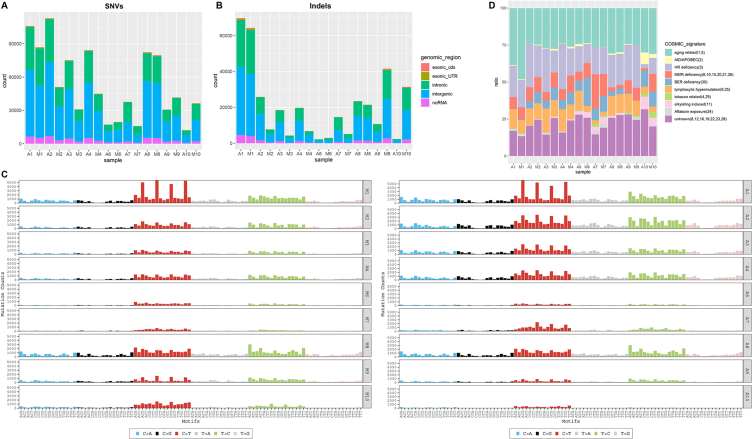


**figs2:**
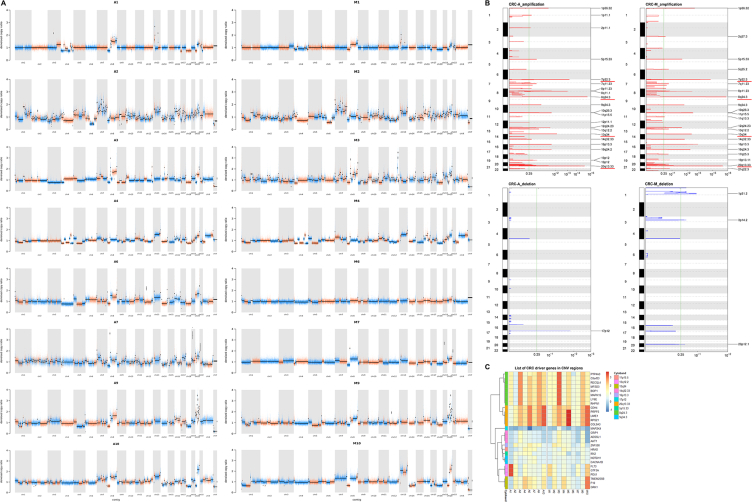


**figs3:**
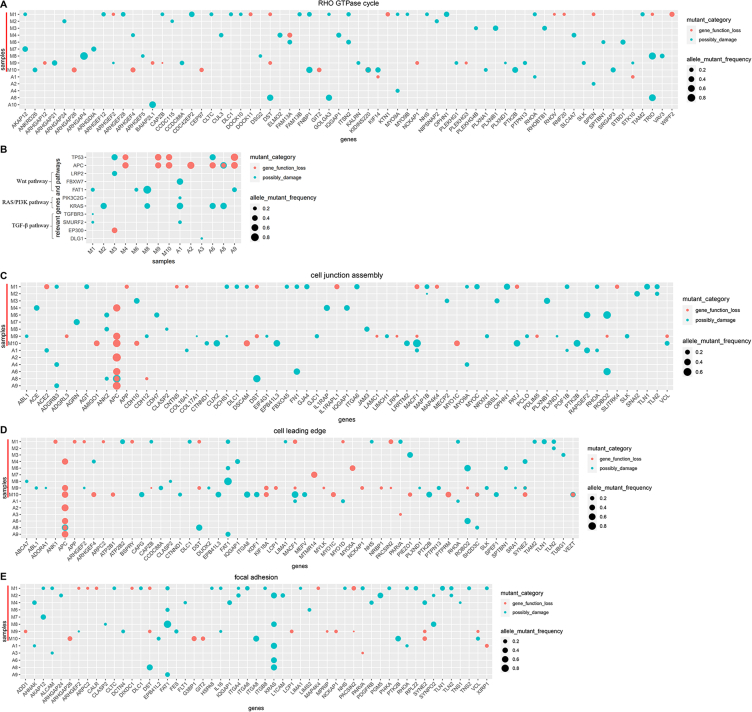


**figs4:**