# Supplementary material for: Investigation of the genome-wide signatures underlying the micropapillary carcinoma components in colorectal cancers
Source: Genes Dis. 2024 May 19;12(2):101331. doi: 10.1016/j.gendis.2024.101331 (PMC11616049; doi:10.1016/j.gendis.2024.101331)
Supplement: Multimedia component 1 [file mmc1.docx]

**Investigation of the genome-wide signatures underlying the micropapillary carcinoma components in colorectal cancers**

**Supplementary information**

**Materials and methods**

**Patients and specimens**

The documented specimens enrolled in this study were collected from the patients diagnosed with colorectal cancer at Nankai University affiliated Union Medical Center of Tianjin, China. The study was approved by the medical ethics committee of Nankai University affiliated Union Medical Center (approval number: 2014KZ058) and we have obtained informed consent from the patients. Procedures involving the use of human pathological materials were in accordance with the Helsinki Declaration of 1975. Sample collection in the study followed the criteria that patients were initially diagnosed and received no other treatment before surgery. Pathological diagnosis of resected samples was given by two senior pathologists independently, particularly for the definite diagnosis of the micropapillary carcinoma (MPC) components. A cohort of 10 patients’ specimens containing typical MPC components were ultimately included in this study, and generally clinical and pathologic information of each patient were listed in Supplementary Table.1. For each specimen, concomitant micropapillary and adenocarcinoma components (abbreviated as CRC-M and CRC-A, respectively), as well as their paracancerous tissue (CRC-P), were dissected by micro-perforation on paraffin-embedded tissue according to their relative positions under microscope (CRC-A component in patient 5 and CRC-P component in patient 8 were lacking).

**Hematoxylin-eosin (HE) and immunofluorescent (IF) staining**

The formalin-fixed and paraffin-embedded (FFPE) tissues were sectioned into 4-μm-thick slices for HE and IF staining. For HE staining, slides were stained with hematoxylin for 2 min, followed by 0.5% hydrochloric acid alcohol differentiation for 10 sec, ammonia anti-blue for 10 sec, eosin staining for 2 min, dehydrate transparent and neutral gum sealing. For IF staining, after rehydrating and antigen retrieval, slides were blocked in 5% normal goat serum for 1 h and incubated with an anti-CD56 antibody (Santa Cruz, sc-7326) and an anti-KLRC1 antibody (Affinity, DF4808) at 4℃ overnight, followed by incubation with fluor-488 and fluor-546 labeled secondary antibodies (CST, 4409S, 4412S) for 1h. Slides were finally sealed with neutral gum containing DAPI and images were collected by a laser confocal microscope (Olympus, Center Valley, PA).

**DNA extraction and** **whole genome sequencing of FFPE samples**

DNA extraction from FFPE samples was performed with QIAamp DNA FFPE Tissue Kit (QIAGEN Shenzhen, China). DNA library construction and whole genome sequencing (WGS) were manipulated by Novogene Co, Ltd (Beijing, China). In brief, genomic DNA fragments with the length ranging from 300bp-500bp were subjected to library construction, and PE150 strategy and Illumina Hiseq-2000 platform was adopted for sequencing. The paired CRC-M, CRC-A and CRC-P samples from each patient were sequenced independently, and clean reads of each sample were obtained by filtering raw data using FASTX-Toolkit software (v.0.0.13). The sequencing depth of each sample reached to 30×. All clean data in fastq format were available at China National Center for Bioinformation (CNCB, <https://ngdc.cncb.ac.cn/hgrip/>, accession number: HRA003804).

**Genomic variation calling and annotation**

The valid sequencing data were mapping to the human reference genome hg38 using BWA algorithm (0.7.8-r455) to obtain the alignment results in BAM format. The BAM files were processed with GATK and samtools for duplicate marking, local realignment, and base quality correction to obtain the final alignment results in BAM format. Somatic mutation calling of each CRC-M and CRC-A sample was performed with the GATK’s Mutet2 pipeline. All paracancerous (CRC-P) samples were used to create a panel of normals (PoN). In the following manipulations, CRC-M and CRC-A samples with matched CRC-P sample were subjected to the “Tumor with matched normal” mode, while CRC-M and CRC-A samples from patient 8, which lacked the CRC-P data, were subjected to the “Single tumor sample” mode using the PoN as the normal_panel input. Algorithms for other steps used default parameters, and estimating and filtering of sequence context artifacts for FFPE samples were employed. Annotation of SNP, InDel and CNV were performed using ANNOVAR (2017June8).

**TCGA data collection and analysis**

TCGA colon carcinoma (COAD) and rectal carcinoma (READ) datasets including clinical information, whole-exome somatic mutation data, copy number variation data, transcriptome data, miRNA expression data and genomic methylation data (level 3) were downloaded from the Cancer Genome Atlas (TCGA, <https://portal.gdc.cancer.gov>). The patient sample ID was used as the unique tag for matching the data of each sample across different datasets. For transcriptome/miRNA expression analyses, genes with the maximum FPKM/TPM value less than 1 in datasets were marked as very-low expressed genes and excluded for further analyses. All data were manipulated and plotted with R software (<http://www.r-project.org>).

**Other bioinformatical analysis**

Mutational signature analyses based on SNVs were performed with R package “deconstructSigs” [1] and “SomaticSignatures” [2] following the standard pipeline. Copy number variation (CNV) analyses to identify the genomic regions with significant gains or losses across bulk samples were performed with GISTIC2.0. All CRC-M and all CRC-A samples were dealt as a group, respectively. The CNV thresholds were defined as: deletion (log ratio < −1.3), loss (−1.3 ≤ log ratio ≤ −0.1), normal (−0.1 < log ratio < 0.1), gain (0.1 ≤ log ratio ≤ 0.9) and amplification (log ratio > 0.9). Gene function enrichment analyses were performed with R package “clusterProfiler” [3] and “ReactomePA” [4]. All statistical analyses were performed using R software and images were primarily drawn by R package ggplot2.

**Supplementary References**

1. Rosenthal R, Mcgranahan N, Herrero J, et al. DeconstructSigs: delineating mutational processes in single tumors distinguishes DNA repair deficiencies and patterns of carcinoma evolution. Genome Biol, 2016, 17: 31.
2. Gehring JS, Fischer B, Lawrence M, et al. SomaticSignatures: inferring mutational signatures from single-nucleotide variants. Bioinformatics, 2015, 31(22): 3673-5.
3. Yu G, Wang L G, Han Y, et al. clusterProfiler: an R package for comparing biological themes among gene clusters. Omics, 2012, 16(5): 284-7.
4. Yu G, He QY. ReactomePA: an R/Bioconductor package for reactome pathway analysis and visualization. Mol Biosyst, 2016, 12(2): 477-9.

**Supplementary Tables**

**Supplementary Table.1 clinical and pathological information of colorectal cancer patients enrolled in the cohort**

| Patient No. | Gender | Age at diagnosis | Pathologic diagnosis |
| --- | --- | --- | --- |
| 1 | F | 48 | Rectal tumor; adenocarcinoma, middle differentiated; MPC component, > 50% |
| 2 | M | 63 | Rectal tumor; adenocarcinoma, low differentiated; MPC component, > 70% |
| 3 | M | 50 | Sigmoid colon tumor; low differentiated, invasive micropapillary carcinoma, MPC component, 80% |
| 4 | M | 79 | Rectal tumor; adenocarcinoma, middle-to-low differentiated; MPC component, 50% |
| 5 | M | 63 | Rectal tumor; adenocarcinoma, middle differentiated; MPC component, 2/3 |
| 6 | F | 54 | Cecal tumor; adenocarcinoma, middle-to-low differentiated; MPC component, > 50% |
| 7 | F | 67 | Rectal tumor; adenocarcinoma, middle-to-low differentiated; MPC component, > 50% |
| 8 | F | 72 | Metastatic adenocarcinoma nodule with focal MPC; MPC component, 50%; |
| 9 | M | 62 | Rectal tumor; adenocarcinoma, middle-to-low differentiated; MPC component, 50% |
| 10 | M | 86 | Rectal tumor; adenocarcinoma, focal MPC; MPC component, 50% |

Sup**plementary Table.2 The list of genes that were identified as MPC-specific function-loss and enriched in cell adhesion and cell motility related functions**

| Genes |  |  |  |  |  |
| --- | --- | --- | --- | --- | --- |
| ADD1 | CDH10 | ERCC6L | LRP4 | MYO3A | SLITRK4 |
| ADGRL3 | CEACAM1 | IL1RAPL1 | MAP4K4 | MYO5A | UNC13B |
| AMIGO1 | CEP97 | KCNQ3 | MYH3 | NCKAP1 | UNC13C |
| APP | CKAP5 | KIF18A | MYH8 | PATJ | VCL |
| ARPC2 | CNTN5 | KTN1 | MYLK | PDLIM5 | WIPF2 |
| ATP2B1 | DSCAM | LAMC1 | MYO1C | SLC8A3 |  |

**Supplementary Table.3 The list of genes that were identified as MPC-specific function-loss and curated driver genes in colorectal cancers**

| Genes |  |  |  |
| --- | --- | --- | --- |
| CHEK2 | ZNF551 | PIAS2 | MIPOL1 |
| RHOV | CDH10 | CEACAM5 | TMTC4 |
| WRN | EP300 | ITPR1 |  |

**Supplementary figure legends**

**Figure.S1** **The overall SNV and Indel profiles of paired micropapillary carcinomas and adenocarcinomas in the enrolled colorectal cancer cohort.** (A-B) Stacked bar plots showing the distribution of single nucleotide variations (SNVs, A) and small insertion-deletions (Indels, B) detected in each sample on defined genomic regions including coding region (cds), UTR, intron, intergenic and non-coding RNA region. M, micropapillary carcinoma; A, adenocarcinoma. (C) Bar plots showing the distribution of the 96-element mutational signatures based on mutated trinucleotides (containing six classes of base substitution: C > A, C > G, C > T, T > A, T > C and T > G) in micropapillary carcinoma (CRC-M, left) and adenocarcinoma (CRC-A, right) samples groups. (D) A spiny diagram showing the distribution of weighted COSMIC signatures in each CRC-M and CRC-A samples. Each color represented a certain term of COSMIC signatures and detailed information was given by the legend.

**Figure.S2** **The overall copy number variation (CNV) profiles of micropapillary carcinomas and adenocarcinomas in the enrolled colorectal cancer cohort.** (A) Plots showing the distribution of denoised CNVs detected in each adenocarcinoma (CRC-A, left) and micropapillary carcinoma (CRC-M, right) samples. Paired CRC-A and CRC-M plots were arranged in parallel. (B) Plots showing the results of GISTIC analysis on CRC-A (left) and CRC-M (right) groups. Plots of CNV amplification and CNV deletion were drawn in red and blue, respectively. *q*-values were marked at the bottom of each plot, and the most significant common CNV regions between CRC-A and CRC-M were indicated by redlines. Rather similar CNV profiles were detected in general (B) and in each paired MPC and adenocarcinoma samples (A) except a few cases (sample 6 and 7). (C) A heatmap listing the curated driver genes in colorectal cancers locating in the CNV regions identified in (B). Different copy number alterations were marked by colors and the genomic location of each gene was indicated in the legend. Although the amplification of a set of proto-oncogenes, such as MAPK15, AKT1, HRAS and NOTCH1, were identified, no specific functions were enriched on by this set of genes.

**Figure.S3 Details of the detected mutant genes enriched in relative terms in Pathway and Gene Ontology databases in the incorporated colorectal cancer samples in this study.** (A) A bubble plot showing the distribution and details of mutant genes enriched in the term RHO-GTPase cycle in the sample cohort. (B) A bubble plot showing the distribution of most frequently mutant genes in each sample in the cohort. (C-E) Bubble plots showing the distribution and details of mutant genes enriched in cell junction assembly (C), cell leading edge (D) and focal adhesion (E) in the sample cohort. The mutant category and mutant frequency were indicated by the markers shown in the legends. Much more mutations were detected in MPC than in adenocarcinoma components as shown in A, C, D and E.

**Figure.S4 Additional information concerning to the TCGA colorectal cancer datasets.** (A) A sketch map showing the strategy for screening of the candidates with MPC components (MPC-signature) in TCGA colorectal carcinoma cohort. A sample set containing 22 samples (the MPC group) was identified. (B-C) Dotplots showing the results of Gene Ontology enrichment assay performed with genes in relative MPC-upregulated gene clusters (B) and MPC-downregulated gene clusters (C). The MPC_up group includes cluster 1, 2, 6, 14 and 10, and the MPC_down group includes cluster 4, 5 and 8. Other information was marked on each plot. (D) Dot and box plots giving the distribution of expression levels of KLRC1, NCAM1 (CD56), HLA-E and HLA-G in MPC, non-MPC and normal tissue groups in TCGA colorectal cancer dataset. ANOVA followed by TukeyHSD test, ***, *p* < 0.001; ns, not significant. (E) Heatmap showing the clustering result of the top 500 differentially methylated sites between the MPC and non-MPC group in TCGA colorectal cancer methylation dataset. (F) Heatmap showing the clustering result of the top 500 differentially expressed miRNAs between the MPC and non-MPC group in TCGA colorectal cancer miRNA expression dataset. Relative information was marked on the plots.
